# Supplementary material for: Analysis of subcellular RNA fractions demonstrates significant genetic regulation of gene expression in human brain post-transcriptionally
Source: Sci Rep. 2023 Aug 24;13:13874. doi: 10.1038/s41598-023-40324-0 (PMC10449874; doi:10.1038/s41598-023-40324-0)
Supplement: Supplementary file 1 — Supplementary Information 1. [file 41598_2023_40324_MOESM1_ESM.pdf]

Analysis of subcellular RNA fractions demonstrates significant genetic regulation of gene expression in human brain post-transcriptionally

Karishma D'Sa[1,2], Sebastian Guelfi[1], Jana Vandrovcova[3], Regina H. Reynolds[4], David Zhang[4], John Hardy[1,5], Juan A. Botía[4,6], Michael E. Weale[2], Sarah A. Gagliano Taliun[7,8,9], Kerrin S. Small[†10] and Mina Ryten[†4,11,*]

[1]Department of Neurodegenerative Disease, University College London, London, WC1N 3BG, UK.

[2]Department of Medical & Molecular Genetics, School of Medical Sciences, King's College London, Guy's Hospital, SE1 1UL, London, UK.

[3]Dept of Neuromuscular Disease, UCL Queen Square Institute of Neurology, London, WC1N 3BG United Kingdom

[4]Great Ormond Street Institute of Child Health, Genetics and Genomic Medicine, University College London, London, WC1N 1EH UK.

[5]UK Dementia Research Institute at University College London, London, WC1N 3BG, UK.

[6]Departamento de Ingeniería de la Información y las Comunicaciones, Universidad de Murcia, Murcia, 30100, Spain.

[7]Department of Medicine, Université de Montréal, Montréal, QC, H3T 1J4, Canada.

[8]Montréal Heart Institute, Montréal, QC, H1T 1C8, Canada.

[9]Department of Neurosciences, Université de Montréal, Montréal, QC, H3T 1J4, Canada.

[10]Department of Twin Research and Genetic Epidemiology, King's College London, London, SE1 7EH, UK.

[11]NIHR Great Ormond Street Hospital Biomedical Research Centre, University College London, London, WC1N 3JH, UK

* To whom correspondence should be addressed. Tel: +44 (0) 20 7242 9789; Email: mina.ryten@ucl.ac.uk

† Joint Authors

Present Address:
Karishma D'Sa, Department of Clinical and Movement Neurosciences, University College London, London, WC1N 3BG, UK.

Sebastian Guelfi, Verge Genomics, Tower Pl, South San Francisco, CA 94080, United States

Michael E. Weale, Genomics plc, Oxford, OX1 1JD, UK.

Supplementary figures

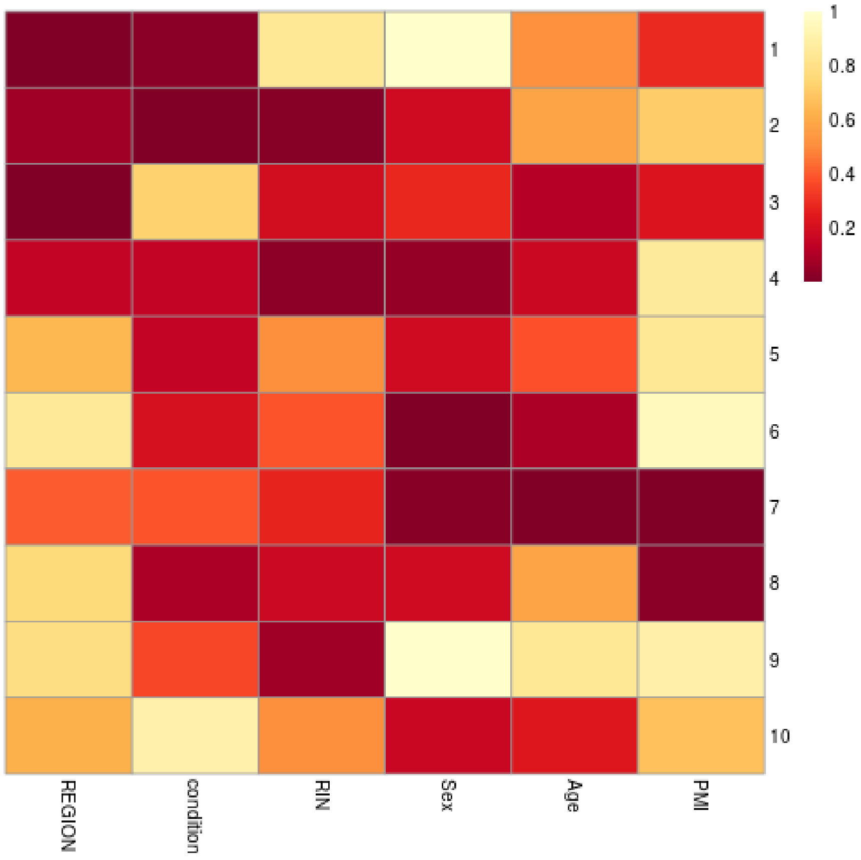

Supplementary figure 1. Principal component analysis plot–Heatmap showing the correlation of the 1st 10 PCs with region, condition, RIN, sex, age and post mortem interval(PMI).

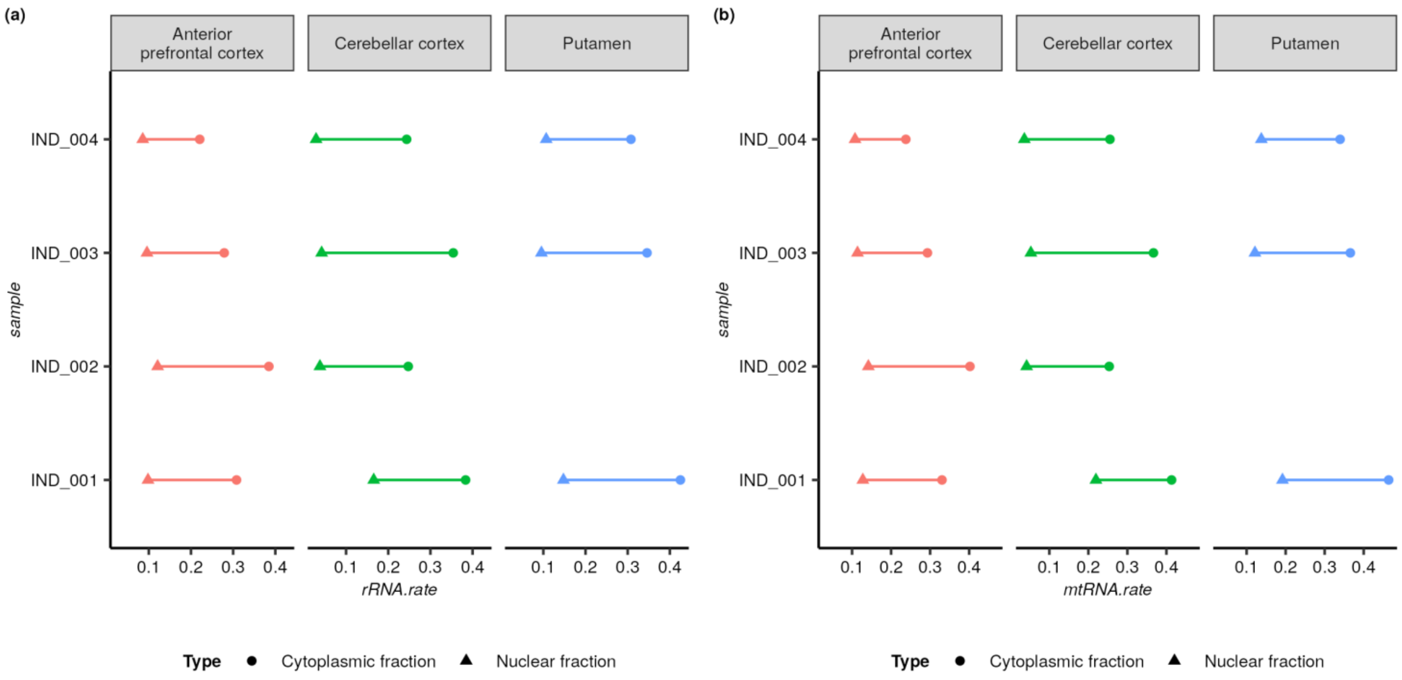

Supplementary figure 2. Assessing quality of fractionation by examining the (a) rRNA rate (b) mtRNA mapping rate in the nuclear vs cytoplasmic fractions.

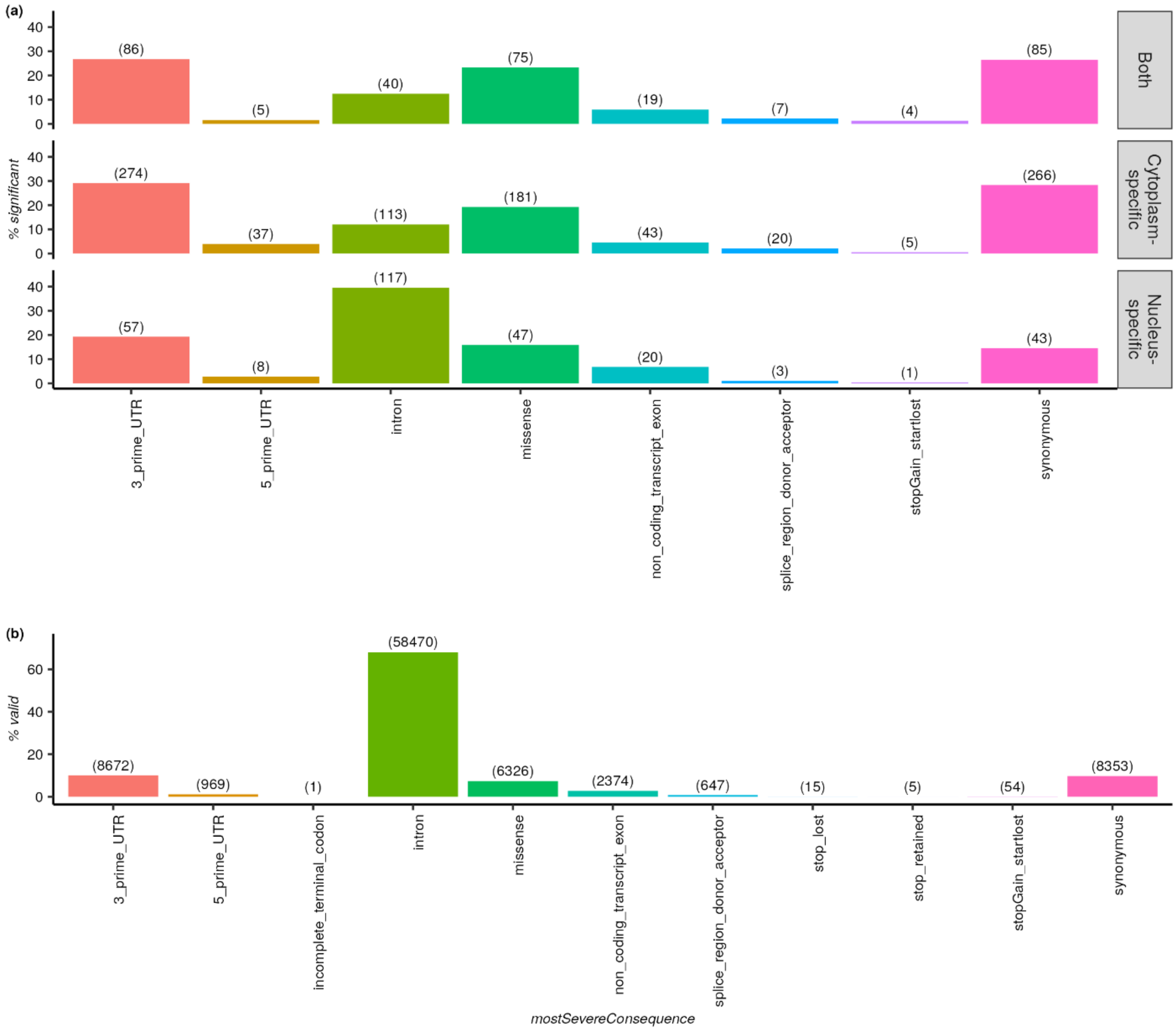

Supplementary figure 3. Distribution of the most severe consequence of heterozygous SNPs (a) with an ASE signal in both fractions or fraction-specific (b) valid in at least 1 fraction

Supplementary tables

Individual ID	Sex	Age	Post-mortem interval in hours
IND_001	Male	57	113
IND_002	Male	50	41
IND_003	Male	53	53
IND_004	Female	41	50

Supplementary table 1. Demographic details of individuals

Tissue	Fraction	Biotypes %		
		Protein coding	lncRNA	Pseudogene
Anterior prefrontal cortex	Cytoplasm	76.8 (4424)	14.9 (114)	15.6 (34)
	Nucleus	23.2 (1339)	85.1 (653)	84.4 (184)
	p-value	< 2.2E-16	2.32E-84	3.01E-24
Cerebellar cortex	Cytoplasm	80.1 (6310)	8.4 (197)	13.4 (58)
	Nucleus	19.9 (1569)	91.6 (2136)	86.6 (374)
	p-value	< 2.2E-16	< 2.2E-16	3.49E-52
Putamen	Cytoplasm	80.5 (5198)	22.1 (161)	22.8 (42)
	Nucleus	19.5 (1256)	77.9 (567)	77.2 (142)
	p-value	< 2.2E-16	3.14E-51	1.46E-13

Supplementary table 2. Percentage of genes with a significant DGE by biotype, in the nucleus and cytoplasm (number of genes are shown within brackets).

Note: The p-values are derived from a 1-sample proportions test with continuity correction as implemented by the prop.test() function in R.

Fraction in which ASE signal detected	Biotype (%)			
	**Protein coding**	**lncRNA**	**Pseudogene**	**Other**
Both fractions	89.3 *(233)*	5.4 *(14)*	0.4 *(1)*	5.0 *(13)*
Cytoplasm-specific	92.9 *(575)*	4.2 *(26)*	0.8 *(5)*	2.1 *(13)*
Nucleus-specific	85.8 *(133)*	9.7 *(15)*	0.0 *(0)*	4.5 *(7)*

Supplementary table 3. Distribution of the biotype of genes with an ASE signal in both fractions or fraction-specific (number of genes are shown within brackets).

Fraction in which ASE signal detected	Most severe consequence (%)		
	**Exon variant**	**Intron variant**	**UTR variant**
Both fractions	57.7 *(179)*	12.9 *(40)*	29.4 *(91)*
Cytoplasm-specific	53.6 *(490)*	12.4 *(113)*	34.0 *(311)*
Nucleus-specific	37.7 *(110)*	40.1 *(117)*	22.3 *(65)*

Supplementary table 4. Distribution of the most severe consequence of ASE signals by the fraction they were significant in (number of ASE signals are shown within brackets).

Tissue	Fraction in which ASE signal detected	Pearson correlation
**Anterior prefrontal cortex**	both fractions	0.99
	cytoplasm-specific	0.79
	nuclear-specific	0.84
**Putamen**	both fractions	0.99
	cytoplasm-specific	0.85
	nuclear-specific	0.80
**Cerebellar cortex**	both fractions	0.98
	cytoplasm-specific	0.75
	nuclear-specific	0.71

Supplementary table 5. Pearson correlation between the nuclear and cytoplasmic allelic ratios for the ASEs in both fractions, cytoplasm and nuclear specific combining across tissues and by tissue.

Source	Term name	Term size	Term id	p-value	Intersection size	Intersection
REAC	CREB1 phosphorylation through NMDA receptor-mediated activation of RAS signaling	19	REAC:R-HSA-442742	2.70E-02	4	ENSG00000117676,ENSG00000145349, ENSG00000132535,ENSG00000176884
REAC	Ras activation upon Ca2+ influx through NMDA receptor	15	REAC:R-HSA-442982	4.74E-02	3	ENSG00000145349,ENSG00000132535, ENSG00000176884
REAC	Negative regulation of NMDA receptor-mediated neuronal transmission	14	REAC:R-HSA-9617324	4.74E-02	3	ENSG00000145349,ENSG00000132535, ENSG00000176884
REAC	Unblocking of NMDA receptors, glutamate binding and activation	16	REAC:R-HSA-438066	4.74E-02	3	ENSG00000145349,ENSG00000132535, ENSG00000176884
REAC	Long-term potentiation	16	REAC:R-HSA-9620244	4.74E-02	3	ENSG00000145349,ENSG00000132535, ENSG00000176884
REAC	Neurotransmitter receptors and postsynaptic signal transmission	114	REAC:R-HSA-112314	4.74E-02	8	ENSG00000145863,ENSG00000117676, ENSG00000176533, ENSG00000109158, ENSG00000145349,ENSG00000132535, ENSG00000176884,ENSG00000162989

Supplementary table 6. Enrichment analysis - significant terms observed for hetSNPs with a significant difference in allelic ratios and with cytoplasmic-only ASE signals
